# 

**Published:** 1907-12-15

**Authors:** 


CONTENTS

Event and Comment - - - - - - - - 593

Real Remoldable Porcelain including Moldable Gold, Silex, Carborundum, Corundum, Clays, etc., *By Chas. H. Land, L.D.S.* 595

The Cast Inlay, - - - - *By W. H. Taggart, D.D.S.* 600

Business Methods, - - - - *By J. H. Irwin, D.D.S.* 605

Brightening Orthodontic Appliances, - *By W. J. Brady, D.D.S.* 613

Indents - - - - - - - - - - - - 617

Announcements - - - - - - - - - - 636

